# Characteristics chloroplast genome of Yangxincai and correction of its Latin scientific name

**DOI:** 10.1080/23802359.2025.2593156

**Published:** 2025-11-26

**Authors:** Ke Zhang, Feng Yang, Hongfei Liang, Chong Luo

**Affiliations:** ^a^ School of Life Sciences, Guizhou Normal University, Guiyang, China; ^b^ College of Teacher Education, Guizhou Normal University, Guiyang, China

**Keywords:** *Phedimus aizoon* L., *Phedimus kamtschaticus* F., Yangxincai, chloroplast genome, phylogenetic analysis

## Abstract

Yangxincai, a “highcalcium vegetable,” is a hybrid of *Phedimus aizoon* L. and *Phedimus kamtschaticus *(Fisch., 1840), historically misidentified as *P. aizoon* L. owing to their morphological similarity. Uncertainty regarding its parentage has impeded official nomenclature and caused confusion in research. To clarify its maternal origin, we sequenced and assembled its complete chloroplast genome, 151,666 bp in length with a GC content of 37.73%, comprising LSC 83,128 bp, SSC 16,704 bp, and a pair of IRs (25,917 bp). A total of 128 genes were annotated,including 84 proteincoding, 36 tRNA, and 8 rRNA genes. Phylogenetic analysis of the maternally inherited chloroplast genome confirmed P. aizoon as the maternal parent, and we propose the corrected name *Phedimus aizoon *×* kamtschaticus* for this hybrid cultivar.

## Introduction

*Sedum aizoon* L. (Linnaeus, 1753) (syn. *Phedimus aizoon* L.) and *Sedum kamtschaticus* F. (Fisch., 1840) (syn. *Phedimus kamtschaticus*) are commonly considered the scientific Latin names for Yangxincai, with the former being more widely used (Kim et al. 2023). *S. kamtschaticus* F. is a perennial herb in the Crassulaceae family of the order Saxifragales, native to East Asia and widely distributed in China, Japan, Korea, Mongolia, and Russia (Xiong et al. 2020). Previous studies have reported that *S. kamtschaticus* F. exhibits a variety of pharmacological properties, including anti-hypertensive, hemostatic, antibacterial, and anti-inflammatory activities (Xu et al. 2018). Its clinical potential in treating cardiac and cerebral hypoxia, ischemia, brain injury, and other disorders has also been extensively documented (Plastun et al. 2018; Xu et al. 2019). The tender stems and leaves, particularly the top 12–15 cm segments, are edible and commonly used as vegetables. They are free of bitterness and have a pleasant taste, suitable for stir-frying, stewing, soup preparation, and cold dishes. Every 100 g of the edible portion contains approximately 2.1 g of protein, 0.7 g of fat, 1.5 g of crude fiber, 2.8 mg of carotene, 315 mg of calcium, 39 mg of phosphorus, 3.2 mg of iron, and various essential vitamins, making its nutritional value superior to that of many conventional vegetables (Wang 2015). As a result, *S. kamtschaticus* F. is popular both in restaurants and home cooking (Wang et al. 2013; Bian et al. 2018). Similarly, *S. aizoon* L. demonstrates a wide range of biological activities, including antioxidant, anti-fatigue, hemostatic, antimicrobial, sedative, hypnotic, anticancer, anti-inflammatory, and cardioprotective effects (Wang et al. 2024). In fact, Yangxincai is a cultivated hybrid between these two species (Wang 2015). However, due to unclear paternal and maternal origins, its scientific Latin name remains uncertain, leading to confusion in scientific research and agricultural applications.

Chloroplasts are plant organelles that harbor a variety of essential proteins involved in photosynthesis and other metabolic processes (Keeling 2004). The chloroplast genome typically consists of four distinct regions: a large single-copy (LSC) region, a small single-copy (SSC) region, and a pair of inverted repeats (IRs) (Wicke et al. 2011). Compared to nuclear and mitochondrial genomes, chloroplast genomes are smaller and relatively conserved, with lower rates of recombination and nucleotide substitution (Daniell et al. 2016). Phylogenetic analyses based on entire chloroplast genome sequences have provided novel insights and significant advances in our understanding of plant evolutionary history (Asaf et al. 2020). Furthermore, non-coding regions of chloroplast genomes have proven useful for plant species identification (Ngai et al. 2023). To date, the complete chloroplast genomes of several *Sedum* species within Crassulaceae, including *Sedum bulbiferum* and *Sedum tricarpum*, have been published (Chen et al. 2022; Deng et al. 2023). Additionally, chloroplast genome sequences of *Phedimus takesimensis*, *P. kamtschaticus*, *P. aizoon*, and *S. middendorffianum* have been reported (Han et al. 2020; Seo et al. 2020). Despite these advancements, the evolutionary origin, taxonomic classification, and chloroplast genomic features of Yangxincai, a hybrid derived from a cross between *P. aizoon* L. and *P. kamtschaticus* F., remain largely unexplored. This lack of clarity has hindered a comprehensive understanding of the evolutionary relationships between Yangxincai and other species within the genus *Phedimus* Raf.

While recent large-scale phylogenomic studies, such as Kim et al. (2023), have clarified the backbone phylogeny of East Asian *Phedimus*, the precise parental origin of important cultivars like Yangxincai, often misidentified as *S aizoon* L., remains unresolved. To fill this critical data gap, the aims of this study were to: (1) sequence the complete chloroplast genome of Yangxincai to identify its maternal parent; (2) clarify its phylogenetic position among closely related species using whole-plastome data; and (3) formally propose a corrected scientific name Yangxincai (*P. aizoon* × *kamtschaticus*) based on these findings. This research provides a crucial molecular foundation for the accurate classification and future study of this important cultivated plant.

## Materials and methods

**Figure 1.**
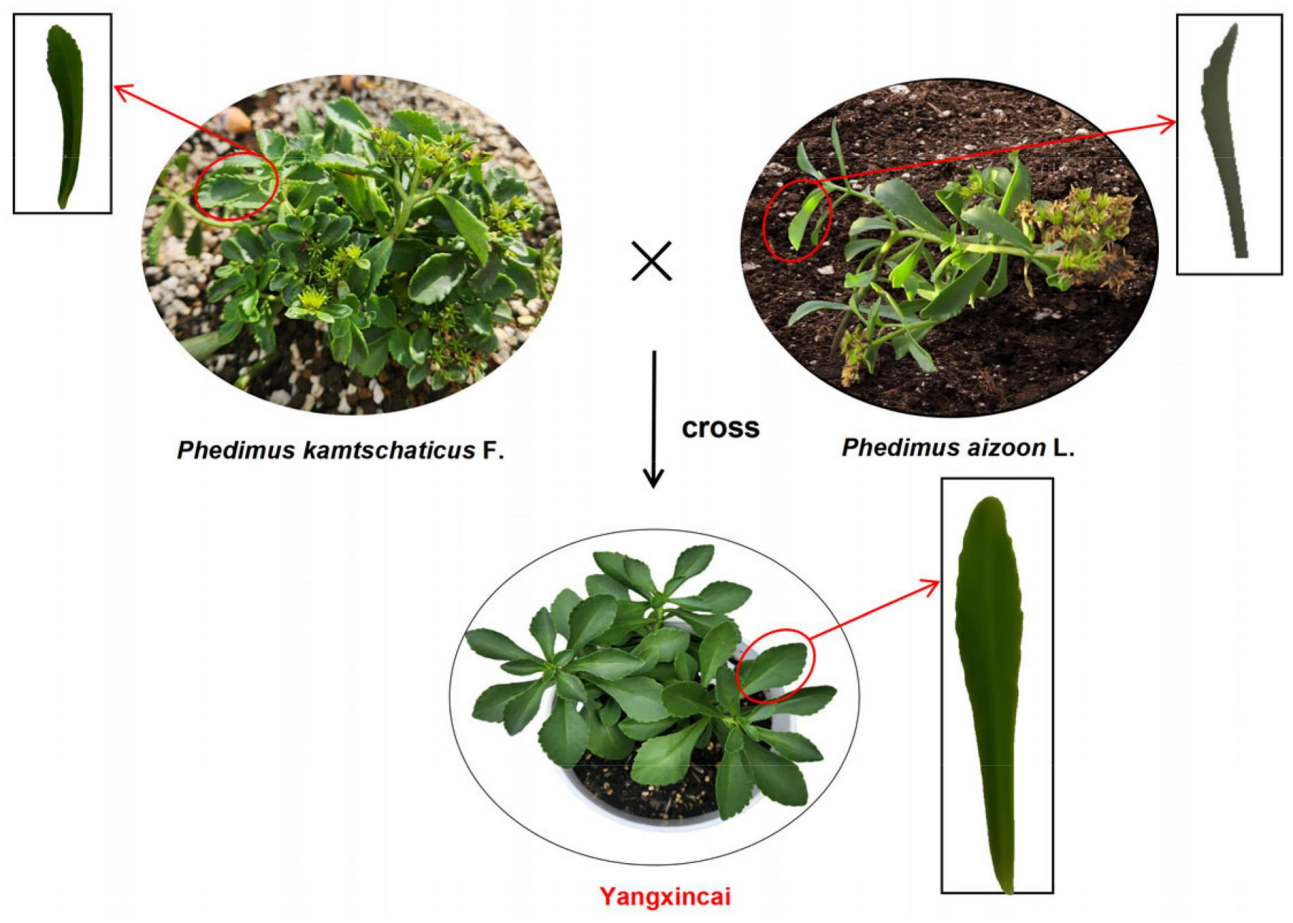
Morphological characteristics of *P. aizoon* L., *P. kamtschaticus* F., and Yangxincai. *P. aizoon* L. exhibits gradually tapering leaf tips that curl downward, *P. kamtschaticus* F. has rounded, blunt leaf apices that curl upward, and Yangxincai possesses rounded, blunt leaf tips that extend horizontally. These photos were taken by the author Ke Zhang at wild vegetable base, Jiuzhou Town, Huangping County, Guizhou Province, China.

Plant samples were collected from a wild vegetable cultivation base located in Jiuzhou Town, Huangping County, Guizhou Province, China (E106.14248, N26.25021; altitude: 684 m) (Figure 1). The plant material was identified as the cultivar Yangxincai by Prof. Luo Chong. Fresh, healthy, and pest-free edible parts were harvested for DNA extraction using the modified CTAB method. The specimen was deposited at the School of Life Sciences, Guizhou Normal University (Contact Person: Ke Zhang; 17802927850m0@sina.cn) under voucher number GZNUZK232200101546.

Total genomic DNA was sequenced on the Illumina NovaSeq 6000 platform (San Diego, CA). After quality control, high-quality reads were used for *de novo* assembly of the chloroplast genome with the GetOrganelle toolkit (Jin et al. 2020). Genome annotation was performed using CPGAVAS2, with the plastome of *P. takesimensis* ‘Chun6’ (GenBank: MZ707524) as the reference. The annotation was subsequently manually curated and refined using CPStools (Huang et al. 2024). The circular genome map was generated with OGDRAW (Greiner et al. 2019), and detailed gene structures were visualized using CPGview (Liu et al. 2023).

To reconstruct the phylogenetic relationships, the newly sequenced Yangxincai plastome was combined with 45 other complete chloroplast genomes from the family Crassulaceae, which were retrieved from the NCBI GenBank database. Two species from a sister genus, *Rhodiola fastigiata* (MN794324) and *Rhodiola subopposita* (OM161977), were selected as the outgroup. The 46 complete plastome sequences were aligned using MAFFT (Katoh and Standley 2013), and the resulting alignment was subsequently trimmed using trimAl (Capella-Gutiérrez et al. 2009) with the -gt 0.8 parameter to remove poorly aligned or divergent regions. Phylogenetic trees were constructed using both maximum-likelihood (ML) and Bayesian inference (BI) methods. For the ML analysis, the best-fit nucleotide substitution model was determined using jModelTest2 (Darriba et al. 2012). The GTR + G + I model was selected and applied for tree construction in RAxML v8 (Stamatakis 2014). Branch support was evaluated through 1000 rapid bootstrap replicates, and the bootstrap support values >50% are shown in the branches. For the BI analysis, MrBayes v3.2.7 (Ronquist et al. 2012) was employed. Two independent Markov chain Monte Carlo (MCMC) runs were performed for 10 million generations, with trees sampled every 1000 generations. The initial 25% of trees were discarded as burn-in, and the remaining trees were used to construct a 50% majority-rule consensus tree to calculate the posterior probabilities (PPs) for branch support.

## Results

**Figure 2.**
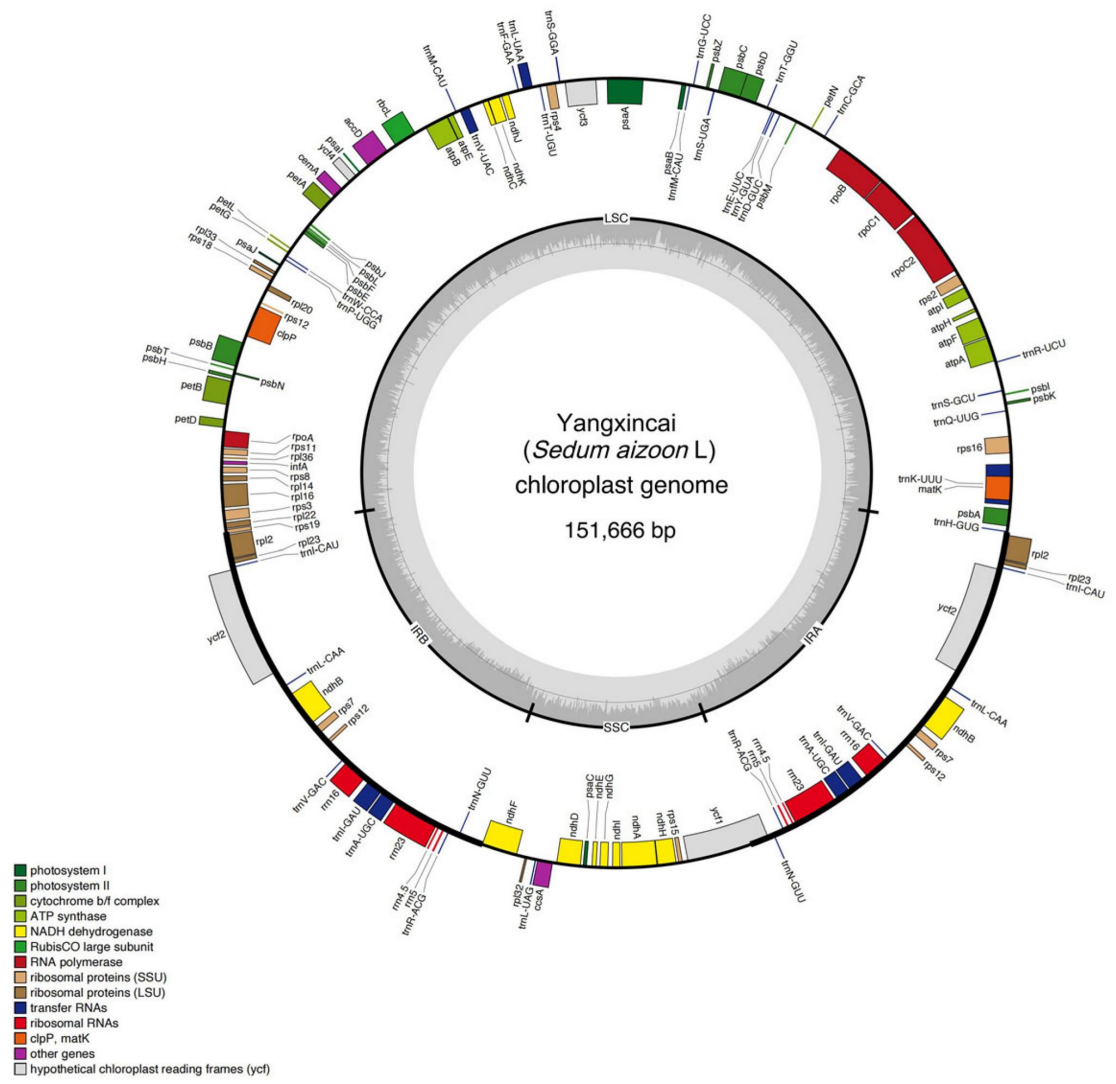
The chloroplast genome map of Yangxincai (*P. aizoon* × *kamtschaticus*). Genes shown outside the outer circle are transcribed clockwise and those inside are transcribed counterclockwise. Genes belonging to different functional groups are color-coded. Dashed area in the inner circle indicates the GC content.

A total of 9.2 GB of clean sequencing data was used for the *de novo* assembly of the chloroplast genome. To verify the accuracy of the assembled genome sequence, we aligned the sequencing reads against the assembled contigs. We obtained a 285× to 7200× depth across the assembled genome, with an average depth of 4020.46× (Figure S1). The results demonstrated the dependability of the assembly result. The final annotated chloroplast genome sequence has been deposited in GenBank under accession number PV528435. The circular gene map is presented in Figure 2, and the detailed gene structure is illustrated in Figure S2.

The complete chloroplast genome of Yangxincai (*P. aizoon* × *kamtschaticus*) is a closed circular DNA molecule with a total length of 151,666 bp. It comprises a typical quadripartite structure consisting of a LSC region of 83,128 bp, a SSC region of 16,704 bp, and two IR regions of 25,917 bp each. The total GC content of the chloroplast genome is 37.73%. The GC content of the LSC region is 35.71%, which is higher than that of the SSC region (31.90%) but lower than that of the IR regions (42.84%). In the chloroplast genome of Yangxincai, 128 functional genes were identified, including 84 protein-coding genes, eight ribosomal RNA (rRNA) genes, and 36 transfer RNA (tRNA) genes, of which 78 protein-coding genes, 29 tRNA genes, and four rRNA genes are unique, respectively. Of these genes, six protein-coding genes (*ndh*B, *rpl*2, *rpl*23, *rps*12, *rps*7, and *ycf*2), seven tRNAs (*trn*I-CAU, *trn*L-CAA, *trn*V-GAC, *trn*I-GAU, *trn*A-UGC, *trn*R-ACG, and *trn*N-GUU), and four rRNAs (*rrn*16, *rrn*23, *rrn*4.5, and *rrn*5) are duplicated in the IR regions. Furthermore, in the chloroplast genome of Yangxincai, nine protein-coding genes (*rps*12, *rps*16, *atp*F, *rpoC*1, *pet*B, *rpl*16, *rpl*2, *ndh*B, and *ndh*A) and six tRNAs (*trn*H-GUG, *trn*K-UUU, *trn*L-UAA, *trn*V-UAC, *trn*I-GAU, and *trn*A-UGC) contained a single intron, while *ycf*3 and *clp*P each contained two introns. In addition, 12 cis-splicing genes and one trans-splicing gene (rps12) were discovered (Figure S2). A comparative analysis revealed that the 151,666 bp plastome of Yangxincai is 273 bp larger than its putative maternal parent, *P. aizoon*, and only 27 bp smaller than its putative paternal parent, *P. kamtschaticus*.

**Figure 3.**
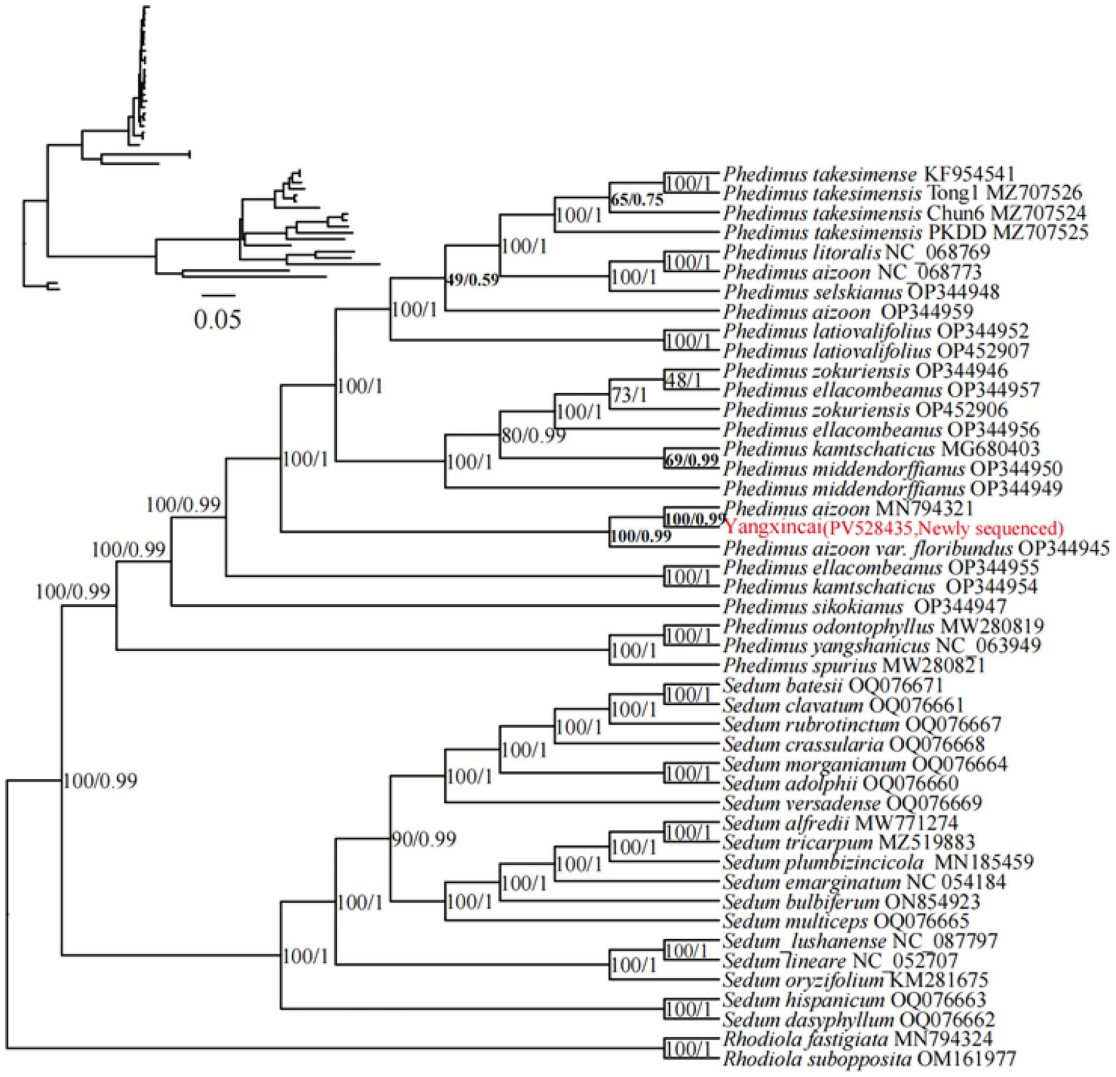
The phylogenetic tree based on the complete chloroplast genomes of 46 representative species of Crassulaceae. The number values at the nodes represent both the maximum-likelihood bootstrap and the Bayesian inference posterior probability. The following sequences were used: *S. emarginatum* (NC 054184, Chang et al. 2020), *S. plumbizincicola* (MN185459, Ding et al. 2019), *S. tricarpum* (MZ519883, Chen et al. 2022), *P. aizoon* (Qinghai-Tibetan Plateau) (MN794321) and *Rhodiola fastigiata* (MN794324, Zhao et al. 2020), *P. kamtschaticus* (MG680403, Seo and Kim 2018), *P. sikokianus* (OP344947), *P. kamtschaticus* (OP344954), *P. ellacombeanus* (OP344955), *P. aizoon var. floribundus* (Korean endemics) (OP344945), *P. middendorffianus* (OP344949), *P. middendorffianus* (OP344950), *P. ellacombeanus* (OP344956), *P. ellacombeanus* (OP344957), *P. zokuriensis* (OP344946), *P. aizoon* (Korean endemics) (OP344959), *P. latiovalifolius* (OP344952), *P. selskianus* (OP344948), *P. litoralis* (NC 068769), *P. aizoon* (Korean endemics) (NC 068773), *P. takesimensis* Chun6 (MZ707524), *P. takesimensis* PKDD (MZ707525), *P. takesimensis* Tong1 (MZ707526) (Kim et al. 2023); *S. hispanicum* (OQ076663), *S. dasyphyllum* (OQ076662), *S. oryzifolium* (KM281675), *S. lushanense* (NC 087797), *S. lineare* (NC 052707), *S. multiceps* (OQ076665), *S. bulbiferum* (ON854923), *S. alfredii* (MW771274), *S. versadense* (OQ076669), *S. morganianum* (OQ076664), *S. adolphii* (OQ076660), *S. crassularia* (OQ076668), *S. rubrotinctum* (OQ076667), *S. batesii* (OQ076671), *S. clavatum* (OQ076661), *P. spurius* (MW280821), *P. yangshanicus* (NC 063949), *P. odontophyllus* (MW280819), *P. zokuriensis* (OP452906), *P. latiovalifolius* (OP452907), *P. takesimense* (KF954541), and *Rhodiola subopposita* (OM161977) are not published; *Rhodiola fastigiata* (MN794324) and *Rhodiola subopposita* (OM161977) were used as an outgroup.

To learn more about the phylogenetic relationship among the members of the *Phedimus* Raf., we built phylogenetic trees using ML and BI methods with the chloroplast genome sequences. Phylogenetic analysis revealed that Yangxincai is more closely related to *P. aizoon* (Qinghai-Tibetan Plateau) (MN794321), one of its parents (Figure 3).

## Discussion and conclusions

Hybridization is a key evolutionary process that generates novel genotypes by combining distinct parental genomes (Arnold and Hodges 1995). It is widely utilized in selective breeding to develop cultivars with desirable traits (Rudolf-Pilih et al. 2019). In nature, inter-specific hybridization may lead to the emergence of new species and underscores the necessity of clearly defining species boundaries (Fitzpatrick et al. 2015). Various approaches have been applied to investigate hybridization events and their impacts on both wild species and cultivated plants (Huamán and Spooner 2002; Huang and Liu 2014; Lema et al. 2019). In Korea, hybrids within the *Phedimus* genus have been reported (Yoo and Park 2016), particularly among the *P. aizoon* subspecies, leading to confusion in distinguishing morphologically similar species such as *P. kamtschaticus* and *P. aizoon* (Han et al. 2020).

In this study, we assembled and analyzed the complete chloroplast genome of Yangxincai (*P. aizoon* × *kamtschaticus*) using Illumina sequencing technology (San Diego, CA). The genome exhibits a typical quadripartite structure with a length of 151,666 bp, containing 128 genes. Its size falls within the known range of *Phedimus* chloroplast genomes, which spans from 147,048 bp to 151,924 bp (Seo et al. 2020; Zhao et al. 2020). Phylogenetic analysis based on complete chloroplast genome sequences revealed that Yangxincai is most closely related to *P. aizoon* (MN794321.1, from the Qinghai-Tibetan Plateau), which is one of its presumed parents. It is followed by *P. aizoon* var. *floribundus* (OP344945. a Korean endemic). In contrast, other *P. aizoon* accessions (e.g. OP344959 and NC_068773) are scattered across the phylogenetic tree, indicating divergence among Korean accessions. The other parental species, *P. kamtschaticus* (e.g. OP344954 and MG680403), clusters together with *P. ellacombeanus* (OP344955) and *P. middendorffianus* (OP344950), forming a distinct clade. Our phylogenetic results support the taxonomic segregation of the genus *Phedimus* from *Sedum*, the largest genus in Crassulaceae. Moreover, Yangxincai clearly groups within the *Phedimus* clade, further confirming its exclusion from the genus *Sedum*. However, unresolved phylogenetic relationships among wild *Phedimus* species still pose challenges in taxonomic classification and cultivar identification.

Based on the fact that the chloroplast genome is inherited through maternal inheritance and the genetic distance between the three species, *P. aizoon* and Yangxincai have the closest genetic distance. Our phylogenetic analysis, combined with the principle of maternal chloroplast inheritance, conclusively identifies *P. aizoon* as the maternal parent of Yangxincai. Accordingly, and in accordance with the naming conventions for hybrids, we formally propose the scientific name *Phedimus aizoon* × *kamtschaticus*. When the maternal parent of a hybrid is unknown, the genetic distance of chloroplast genomes serves as a decisive criterion for its identification. This study provides the first molecular evidence clarifying the hybrid origin of Yangxincai, demonstrating that molecular phylogenetic analysis is a powerful tool for resolving species identification ambiguities and tracing the ancestry of cultivated plants. Furthermore, this case study shows that molecular phylogenetics can effectively resolve longstanding taxonomic controversies and improve species delimitation, as exemplified within the genus *Phedimus*.

## Supplementary Material

Supplementary materials.docx

Proof of selective breeding process for Yangxincai.pdf

## Data Availability

The data of this study are openly accessible in GenBank of NCBI under the accession number PV528435. The associated BioProject, SRA, and BioSample numbers are PRJNA1158565, SRR30607270, and SAMN43543258, respectively.
