# Supplementary material for: Characteristics chloroplast genome of Yangxincai and correction of its Latin scientific name
Source: Mitochondrial DNA B Resour. 2025 Nov 26;10(12):1237–42. doi: 10.1080/23802359.2025.2593156 (PMC12667292; doi:10.1080/23802359.2025.2593156)

**Supplementary fig. 1.** The coverage depth of the complete chloroplast genome of Yangxincai .

1. Total genome length = 151,666 bp (2) Average depth=4026.46 x

(3) Maximaldepth=7200 x (4) Minimal depth=285 x（at the joint）


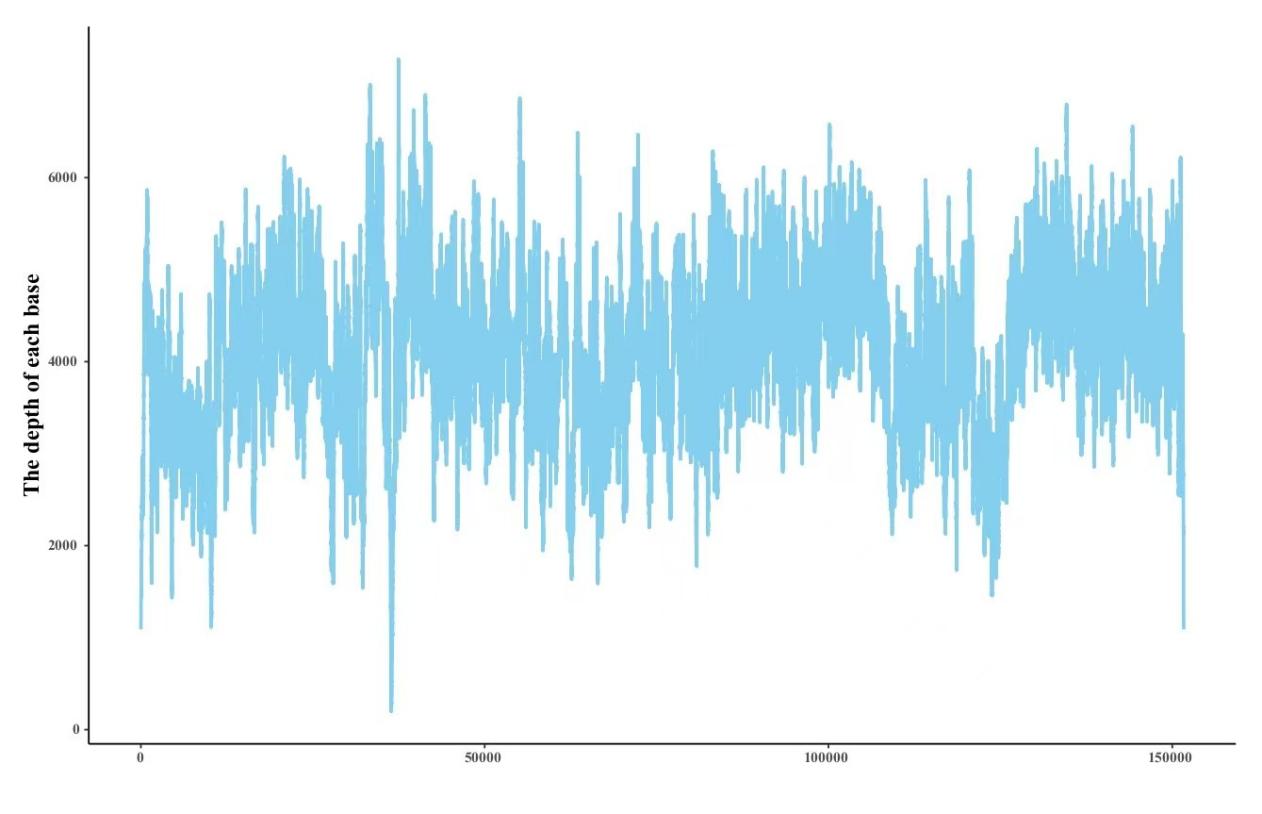


**Supplementary fig. 2.** A schematic map of the cis- and trans-splicing genes of Yangxincai. (A) The schematic map of cis-splicing genes. (B) The schematic map of trans-splicing genes (rps12).

(A)


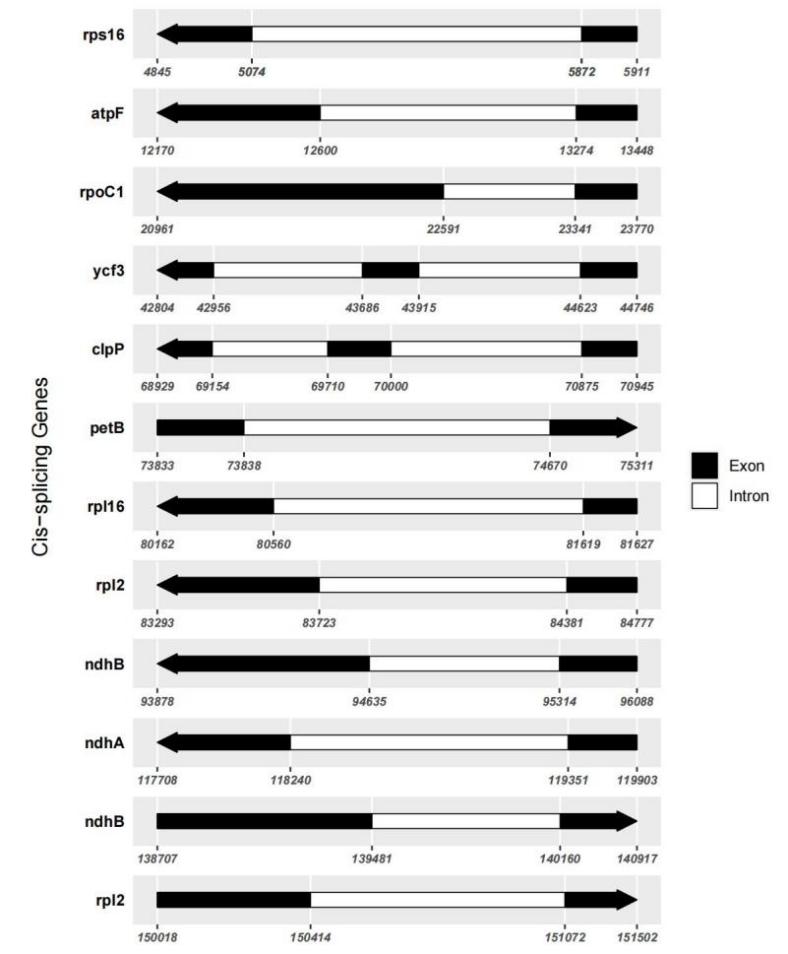


(B)


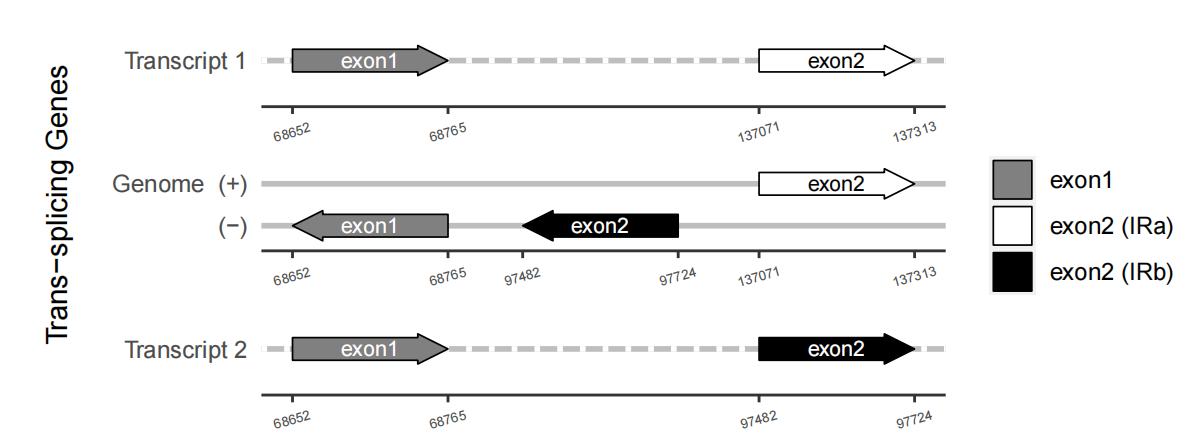

Supplement: Supplementary materials.docx [file TMDN_A_2593156_SM3961.docx]
