# Supplementary material for: Characteristics chloroplast genome of Yangxincai and correction of its Latin scientific name
Source: Mitochondrial DNA B Resour. 2025 Nov 26;10(12):1237–42. doi: 10.1080/23802359.2025.2593156 (PMC12667292; doi:10.1080/23802359.2025.2593156)
Supplement: Proof of selective breeding process for Yangxincai.pdf [file TMDN_A_2593156_SM3960.pdf]

Proof of selective breeding process for Yangxincai

Yangxincai is a new and improved variety bred by crossing *Phedimus aizoon* L. (female parent) with *Phedimus kamtschaticus* Fisch. (male parent). It inherits the superior traits of both parents.

Cultivation Methods and Key Stages

1.The female parent was selected as *Phedimus aizoon* L., and the male parent as *Phedimus kamtschaticus* Fisch.

2.The female parent was planted in a greenhouse to advance its flowering period by 25 days, aligning it with the male parent's flowering period. The two parents were planted in separate nurseries.

3.Timely emasculation was performed during the flowering period of the female parent (*P. aizoon* L.).

4.Pollination of the female parent was conducted using pollen from the male parent (*P. kamtschaticus* Fisch.).

5.Bags were used for isolation and protection after pollination.

6.The plants were maintained until seeds matured, followed by harvesting and threshing.

7.Seedlings were sown and raised in early spring.

8.Growth variations of the seedlings were observed.

9.A certain number of plants with significantly superior growth traits compared to the parents were selected for separate cultivation over one growth cycle.

10.These selected plants were cultivated alongside the parent plants under identical conditions for comparative observation and analysis. Further selection was conducted to retain a small number of plants with stable traits that were significantly superior to the parents, which were then cultivated separately for another cycle.

11.Repeated comparative observations under the same conditions led to the selection of one plant with the most stable traits and comprehensive advantages, which was designated as the optimal hybrid variety.

12.Asexual propagation through cuttings and division was adopted to gradually expand the population. After passing authoritative laboratory tests and meeting the requirements, large-scale asexual propagation was carried out, followed by promotion, application, and production.

Timeline of the Main Breeding Process (6 Years)

June 6–8, 1995: Pollination initiated.

September 2, 1995: 3,500 hybrid seeds harvested.

February 18, 1996: Seeds sown; seedlings emerged from March 3–6 (1,400 seedlings); 880 seedlings survived by May 18; 48 plants with significantly superior traits were selected on September 10.

March 1997: The 48 new varieties and parent plants were planted in three rows on the same plot for comparative growth observation over one year. On April 25, 1998, 6 stable and superior varieties were selected.

May 1, 1998: 6 plants of *P. aizoon* L., 6 plants of *P. kamtschaticus* Fisch., and 6 new varieties were planted on the same plot.

April 28, 1999: One plant was identified as the optimal variety. This plant produced 120 tillers that year, with a height 1.8 times that of *P. kamtschaticus* Fisch. and 1.2 times that of *P. aizoon* L. It maintained stable growth for consecutive years with excellent comprehensive traits.

May 1, 1999: Propagation of this variety began, tentatively named "**Jiuxincai**".

March 20, 2000: A total of 18,000 plants were divided and planted on an area of 0.067 hectares.

Late December 2001: The total number of seedlings reached over 2.8 million.

2002: Promotional articles were published in journals such as Contemporary Agriculture, Agricultural Products Market Weekly, and China Planting Technology. 2003: Reports were published in journals including Yangtze River Vegetables, Beijing Agriculture, New Farmers, and Rural Affairs. May 2004: The variety was renamed "**Yangxincai**". It was tested at the Jiangsu Academy of Agricultural Sciences and the Ministry of Agriculture Wuhan Food Quality Supervision, Inspection and Testing Center. Yangxincai obtained green food certification in many planting bases.

Breeders: Cheng ge Zhang Contact Phone: +86 13585482111
Email: 379235777@qq.com Affiliation: The Wild Vegetable Research Institute of Peixian
Date: August 21, 2025
